# Anti-Obesity Effects of Sulphated Polysaccharides Derived from Marine Macro Algae or Seaweeds: A Systematic Review and Meta-Analysis

**DOI:** 10.3390/md22120528

**Published:** 2024-11-25

**Authors:** S’thandiwe Nozibusiso Magwaza, Vunene Nkateko Chabalala, Nothando Philile Hlongwane, Huda Ismail, Md. Shahidul Islam

**Affiliations:** Department of Biochemistry, School of Life Sciences, University of KwaZulu-Natal (Westville Campus), Durban 4000, South Africa

**Keywords:** animal model, marine macro algae, obesity, seaweeds, sulphated polysaccharides

## Abstract

Sulphated polysaccharides (SPs) are negatively charged compounds found in the cell wall of seaweeds or marine macro algae. These compounds exhibit a range of pharmacological activities, including anti-obesity effects. The aim of this systematic review as well as meta-analysis was to assess the potentials of seaweed-derived SPs to mitigate obesity through a systematic review and meta-analysis of animal model-based studies. A comprehensive summary of the included articles was conducted, focusing on the following obesity-related parameters: food intake, body weight gain, epididymal fat size, adipocyte size, liver weight, serum alanine transaminase (ALT) and aspartate transaminase (AST), insulin and tumour necrosis factor-α (TNF-α), and the lipid profile (total cholesterol, triglyceride, high-density lipoprotein cholesterol (HDL-c), and low-density lipoprotein cholesterol (LDL-c)). The systematic review demonstrated that seaweed-derived SPs exhibit ameliorative effects against obesity, as evidenced by reductions in food intake, body weight gain, epididymal fat and adipocyte size, liver weight, ALT and AST levels, serum insulin and TNF-α, LDL-c, total cholesterol, and triglycerides and an increase in HDL-c in obese rats administered with seaweed-derived SPs. However, the meta-analysis revealed statistically significant anti-obesity effects of seaweed-derived SPs for most, but not all the parameters tested. Further research in human subjects is necessary not only to ascertain the results of preclinical studies but also to provide conclusive evidence of the anti-obesity potential of SPs in humans.

## 1. Introduction

Obesity is one of the major threats to the global public health system. It is classified based on the body mass index (BMI), with a BMI ≥ 25–30 kg/m^2^ indicating overweight and a BMI ≥ 30 kg/m^2^ representing obesity [1]. According to the World Obesity Federation [2], 2.6 billion adults were overweight and obese in 2020, with this figure expected to rise to 6 billion obese adults by 2035, affecting more than half of the global population. Obesity is a complex chronic and metabolic disorder caused by an abnormal or excessive accumulation of fat in the adipose tissue. The metabolic complications of obesity referred to as ‘metabolic syndrome’ include diabetes, hypertension, dyslipidemia, and cardiovascular diseases [1]. Other health conditions include some cancers, mental health illnesses, gallbladder disease, asthma, thyroid dysfunction, and polycystic ovary syndrome or PCOS [3], which are also associated with obesity.

Obesity is caused by an energy imbalance, where there is an excessive energy intake compared to energy usage. This leads to an increase in the accumulation of fats or triglycerides in the white adipose tissue (WAT), an active endocrine organ that stores a significant amount of lipids [4]. Excessive accumulation of fats in the WAT causes hyperplasia (formation of new adipocytes) and hypertrophy (increase in existing adipocyte size), which alter the adipocyte secretome, thereby influencing the nearby microenvironment [5]. This is accompanied by an increased infiltration of macrophages into the adipose tissues from the circulation, where they secrete inflammatory cytokines such as tumour necrosis factor-α (TNF-α), interleukin-6 (IL-6), and interleukin-8 (IL-8). Obese or overweight people have been shown to have elevated inflammatory cytokine levels and may be in a low-grade or chronic inflammation state [6,7]. Inflammation and oxidative stress are closely related pathophysiological processes, and one can readily trigger the other [8]. Inflammation induces oxidative stress through inflammatory cytokines releasing several reactive element species in the adipose tissue. Oxidative stress is an imbalance between the production of reactive oxygen species (ROS) and antioxidants to remove them. The ROS can activate intracellular signalling pathways, increasing the gene expression of proinflammatory cytokines [8], which are closely linked with lipid and carbohydrate metabolism.

The increased consumption of lipids and carbohydrates are potential sources of fats stored in the WAT. This has led to the development of lipid- and carbohydrate-digesting enzyme inhibitors, with promising results in terms of glycemic control and weight loss [9]. Abnormal lipid metabolism (dyslipidemia) is common in obese patients, indicated by elevated triglycerides (TGs), apolipoproteins, very low-density lipoprotein cholesterol (vLDL-c), and low-density lipoprotein cholesterol (LDL-c), with low levels of high-density lipoprotein cholesterol (HDL-c) [10], which are the indications of non-alcoholic fatty liver disease (NAFLD). Evidence has shown that low-carbohydrate diets can improve dyslipidemia [11]. Hence, the first line prevention and treatment of obesity is the consumption of a healthy diet with low calories accompanied by regular exercise [12]. However, many people are unable to maintain this lifestyle due to many reasons. Furthermore, the public need to be aligned with frequently changing and broad dietary recommendations also poses an issue. There are pharmacological treatments for obesity, such as lipase inhibitors and glucagon-like peptide 1 (GLP-1) receptor agonists [13]; however, they have side effects and are costly. Despite of several efforts by healthcare professionals and obese people to treat this disease, they remained dissatisfied with the approach of obesity management. As a result, new and more suitable and effective treatments with less or no side effects are needed to attenuate this disease; natural products and their bioactive compounds are the first choice for this purpose, particularly in the developing world.

Natural products derived from seaweeds have gained traction in biomedical research due to their potential to replace synthetic nutritional supplements and treat a variety of diseases. Many compounds found in marine macro algae or seaweeds have been isolated to use in pharmaceutical products [14]. Sulphated polysaccharides (SPs) are negatively charged polysaccharides found in the cell wall of seaweeds and are biologically active compounds that have garnered significant attention in food, pharmaceutical, and cosmetics industries due to their versatile applications and potential health benefits [15]. Many studies have demonstrated SPs to have promising biological activities including anti-coagulant, anti-hyperglycemic, antioxidant, immunomodulatory, anti-inflammatory, anti- obesity, anticancer, anti-hepatopathy, and anti-viral properties [16,17,18,19]. These findings highlight the significance of exploring SPs as a valuable resource for drug development, contributing substantially to the advancement of biomedical research and the potential treatment of diseases, including obesity.

Based on the above, several studies have reported SPs to have anti-obesity properties; however, there is currently a lack of secondary and comprehensive evidence to support these claims. Therefore, this study aims to conduct a systematic review and meta-analysis on the potential of seaweed-derived SPs to alleviate obesity and related parameters using in vivo preclinical studies, since the numbers of clinical trials with SPs are virtually missing. This will provide a definitive conclusion as to whether these compounds should be isolated and investigated in clinical trials or further investigations should be conducted.

## 2. Results

Forty-four (44) articles were identified during the search and 11 of them were selected for the meta-analysis based on the eligibility and inclusion criteria of the study. In this review, included studies evaluated the effect of SPs, particularly derived from seaweeds, on obesity and related parameters using in vivo obese animal models. The obesity models were primarily rats and mice, where a high-fat diet was used to induce obesity. The SP was administered to the animals using the oral gavage, intraperitoneal, or intragastric method. The data of obesity parameters such as food intake, body weight gain, epididymal fat size, adipocyte size, liver weight, serum ALT, AST, insulin and TNF-α, total cholesterol, total triglyceride, HDL-c, and LDL-c were derived from the articles, as shown in Table 1.

### 2.1. Meta-Analysis on the Effect of Seaweed-Derived SPs Compared to Control Group (Placebo)

#### 2.1.1. Effects of Seaweed-Derived SPs on Food Intake, Body Weight Gain, Epididymal Fat Size, and Adipocyte Size

**Figure 1:**
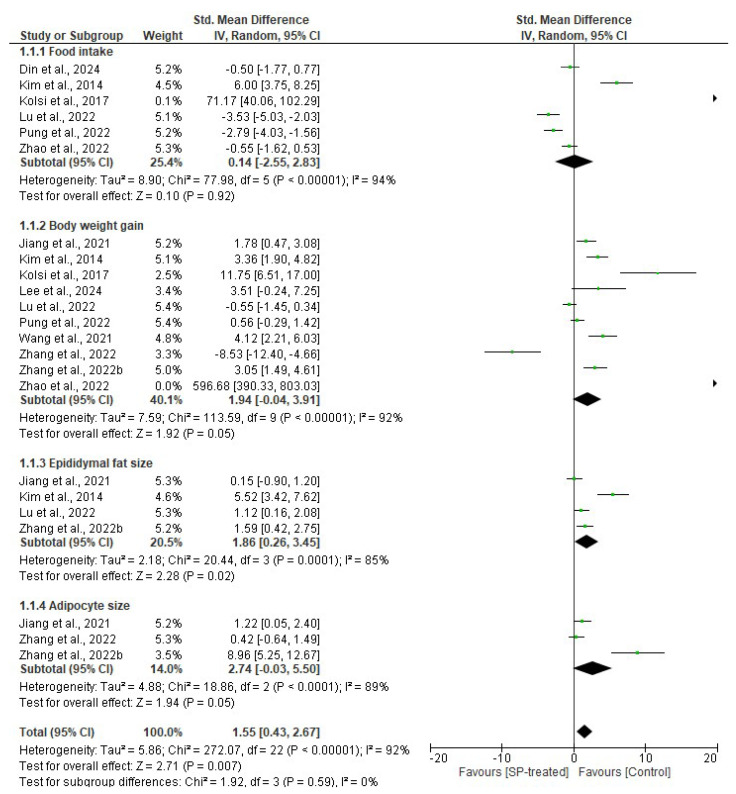
The effect of seaweed-derived SPs on food intake, body weight gain, epididymal fat size, and adipocyte size [18,20,21,22,23,24,25,26,27,28,29]. The forest plot shows the impact of seaweed-derived SP treatment on food intake, body weight gain, epididymal fat, and adipocyte size compared to the control group. The values of *p* ≤ 0.05 were considered significantly different from each other.

**Figure 2:**
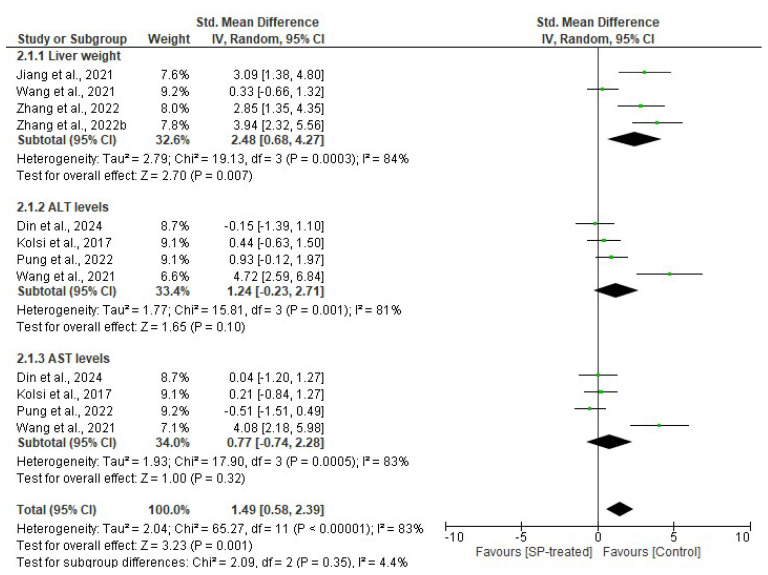
Effects of seaweed-derived SPs on liver weight, serum ALT, and serum AST levels [20,21,23,25,26,27,28]. The forest plot shows the impact of seaweed-derived SP treatment on liver weight, serum ALT, and AST compared to the respective control groups. The values of *p* ≤ 0.05 were considered significantly different from each other.

Figure 1 shows the meta-analysis that reported the effects of SPs on obesity-related parameters including food intake, body weight gain, epididymal fat, and adipocyte size compared to the normal control. Although the analysis revealed no significant difference (*p* = 0.92) between the SP-treated group and the control group in terms of food intake (*n* = 50, SMD = 0.14, 95% CI= −2.55, 2.83), the body weight gain (*n* = 81, SMD = 1.94, 95% CI = −0.04, 3.91), epididymal fat size (*n* = 35, SMD = 1.86, 95% CI = 0.26, 3.45), and adipocyte size (*n* = 22, SMD = 2.74, 95% CI = −0.03, 5.50) were positively and significantly (*p* = 0.05, *p* = 0.02, and *p* = 0.05, respectively) favoured to reduce all these parameters in the SP-treated groups compared to the respective control groups (Figure 2). The heterogeneity for food intake (*I*^2^ = 94%, df = 5 *p* < 0.00001), body weight gain (*I*^2^ = 92%, df = 9 *p* < 0.00001), epididymal fat (*I*^2^ = 85%, df = 3 *p* = 0.0001), and adipocyte size (*I*^2^ = 89%, df = 2 *p* = 0.0001) indicated significant variations on the effect sizes of included studies.

#### 2.1.2. Effects of Seaweed-Derived SPs on Liver Weight and Enzymes 

Four studies reported on the effects of seaweed-derived SPs on liver weight in comparison to their respective controls. There was a significant (*p* = 0.007) reduction (*n* = 33, SMD = 2.48, 95% CI = 0.68,4.27) in liver weight observed in the SP-treated groups compared to the respective control groups and a significant heterogeneity was observed in the included studies (*I*^2^ = 84%, df = 3, *p* = 0.0003). However, four studies were selected for the evaluation of the effects of SPs on the activities of liver function enzymes, ALT and AST, compared to the control. The analysis revealed no significant difference in the ALT (*n* = 28, SMD = 1.24, 95% CI = −0.23, 2.71) and AST (*n* = 28, SMD = 0.77, 95% CI = −0.74, 2.28) levels of the SP-treated groups compared to the respective control groups, with significant heterogeneity *(I^2^* = 81%, df = 3, *p* = 0.001 and *I*^2^ = 83%, df = 3, *p* = 0.0005), as shown in Figure 2.

#### 2.1.3. Effects of Seaweed-Derived SPs on Inflammatory Biomarkers

**Figure 3:**
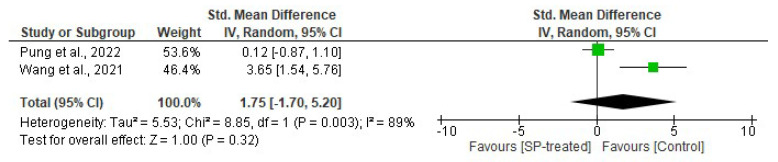
Effects of seaweed-derived SPs on an obesity-related inflammatory biomarker, TNF-α [25,26]. The forest plot shows the impact of seaweed-derived SP treatment on TNF-α compared to the control group. The values of *p* ≤ 0.05 were considered significantly different from each other.

The effects of seaweed-derived SPs on inflammatory biomarkers were evaluated on tumour necrosis factor alpha or TNF-α levels in comparison to the control. There was no significant difference (*n* = 14, SMD = 1.75, 95% CI = −1.70, 5.20) in the inflammatory levels of the SP-treated group and the control. Significant variations (*I*^2^ = 89%, df = 1, *p* = 0.003) were observed in the included studies (Figure 3).

#### 2.1.4. Effects of Seaweed-Derived SPs on Serum Insulin 

**Figure 4:**
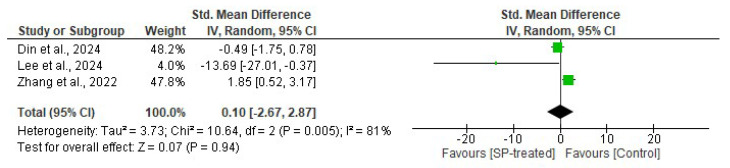
Effects of seaweed-derived SPs on serum insulin [18,20,27]. The forest plot shows the impact of seaweed-derived SP treatment on serum insulin levels compared to the respective control groups. The values of *p* ≤ 0.05 were considered significantly different from each other.

Three studies were included to evaluate the effects of seaweed-derived SPs on serum insulin levels in comparison to their respective controls, and findings are shown in Figure 4. There was no significant difference (*n* = 15, SMD = 0.10, 95% CI = −2.67, 2.87) observed in the serum insulin levels of the SP-treated groups compared to their respective controls. The heterogeneity test revealed significant variations (*I*^2^ = 81%, df = 2, *p* = 0.005) in the included studies.

#### 2.1.5. Effects of Seaweed-Derived SPs on the Serum Lipid Profile

**Figure 5:**
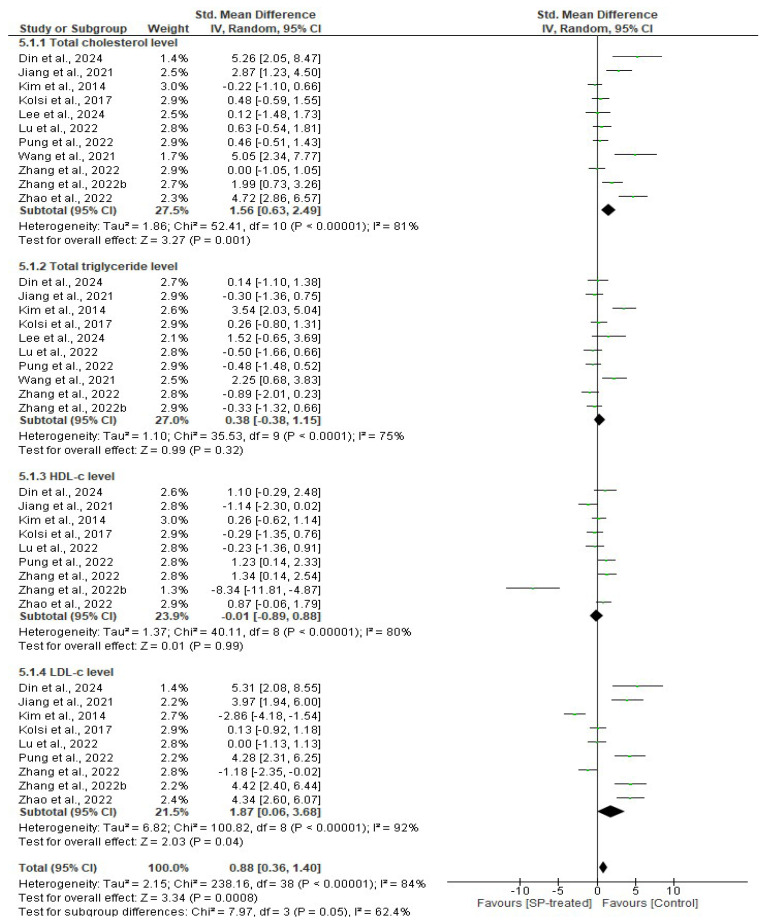
Effects of seaweed-derived SPs on the serum lipid profile, including total cholesterol, triglycerides, LDL cholesterol, and HDL cholesterol [18,20,21,22,23,24,25,26,27,28,29]. The forest plot shows the impact of seaweed-derived SP treatment on the serum lipid profile compared to the untreated group. The values of *p* ≤ 0.05 were considered significantly different from each other.

All studies were included in the evaluation of the seaweed-derived SPs on lipid biomarkers including total cholesterol, triglycerides, HDL-c, and LDL-c in comparison to their respective controls. Eleven, ten, and nine studies assessed the serum total cholesterol, triglycerides, and HDL-c and LDL-c, respectively. There was a significant difference in the total cholesterol (*n* = 77 SMD = 1.56, 95% CI = 0.63, 2.49) and LDL-c (*n* = 68 SMD = 1.87, 95% CI = 0.06, 3.68) levels between the SP-treated group and the untreated group, which means the total cholesterol (*p* = 0.001) and LDL-c (*p* = 0.04) levels were significantly reduced in the SP-consumed groups compared to their respective controls. However, there was no significant difference in the serum triglyceride (*n* = 67 SMD= 0.38, 95% CI= −0.38–1.15) and HDL-c levels (*n* = 68 SMD = 1.87, 95% CI = 0.06, 3.68) in the SP-treated groups compared to their respective control groups (Figure 5). Significant heterogeneity was observed in studies included for total cholesterol (*I*^2^ = 81%, df = 10, *p* < 0.00001), total triglyceride (*I*^2^ = 75%, df = 9, *p* < 0.0001), HDL-c (*I*^2^ = 80%, df = 8, *p* < 0.00001), and LDL-c (*I*^2^ = 92%, df = 8, *p* < 0.00001).

### 2.2. Meta-Analysis on the Effect of Seaweed-Derived SPs in Comparison to the Untreated Group

#### 2.2.1. Effects of Seaweed-Derived SPs on Obesity-Related Parameters

**Figure 6:**
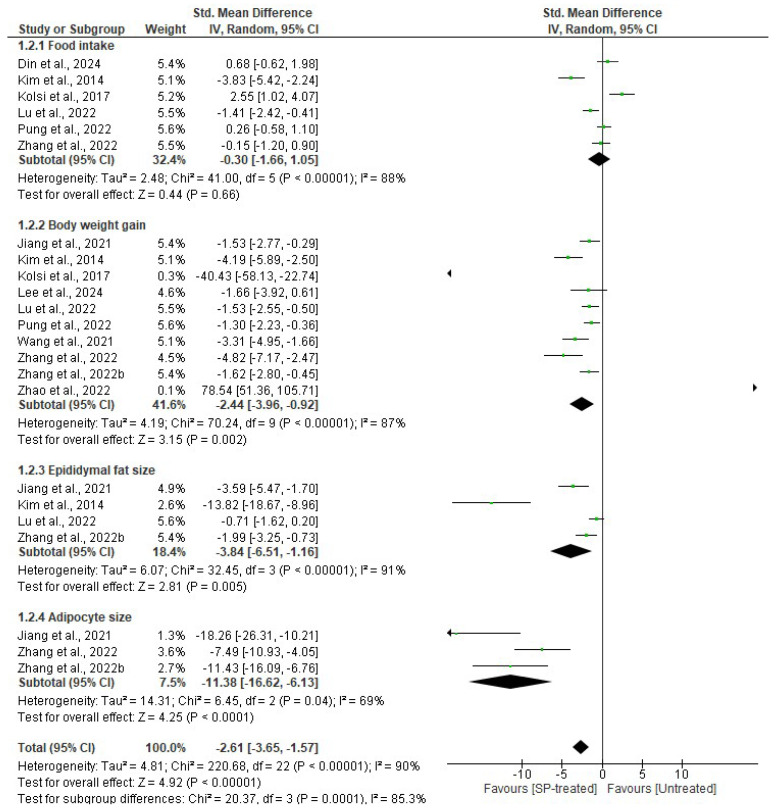
Effects of seaweed-derived SPs on obesity-related parameters such as food intake, body weight gain, epididymal fat size, and adipocyte size [18,20,21,22,23,24,25,26,27,28,29]. The forest plot shows the impact of seaweed-derived SP treatment on obesity parameters compared to the untreated group. The values of *p* ≤ 0.05 were considered significantly different from each other.

Six studies examined the effect of seaweed-derived SPs on food intake in comparison to an untreated group. Although the analysis did not show any significant difference (*n* = 50 SMD = −0.30, 95% CI = −1.66, 1.05) in terms of food intake (*p* = 0.66) in the seaweed-derived SP-treated groups compared to their respective untreated groups (Figure 6), the body weight gain (*n* = 81 SMD = −2.44, 95% CI = −3.96, −0.92), the epididymal fat size, (*n* = 35 SMD = −3.84, 95% CI = −6.52, −1.16), and adipocyte size (*n* = 22 SMD= −11.38, 95% CI = −16.62, −6.13) were significantly reduced (*p* = 0.002, *p* = 0.005, and *p* < 0.0001, respectively) in the SP-treated groups compared to their respective untreated groups. Additionally, the effects were significantly better for the reduction in body weight (*p* = 0.002) and adipocyte size (*p* < 0.0001) compared to the reduction in epididymal fat size (*p* = 0.005). A significant heterogeneity was observed in the studies evaluated for food intake (*I*^2^ = 88%, df = 5, *p* < 0.00001), body weight gain (*I*^2^ = 87%, df = 9, *p* < 0.00001), epididymal fat size (*I*^2^ = 91%, df = 3, *p* < 0.00001), and adipocyte size (*I*^2^ = 69%, df = 2, *p* = 0.04) between seaweed-derived SP-treated and corresponding untreated groups.

#### 2.2.2. Effects of Seaweed-Derived SPs on Liver Weight and Liver Function Enzymes 

**Figure 7:**
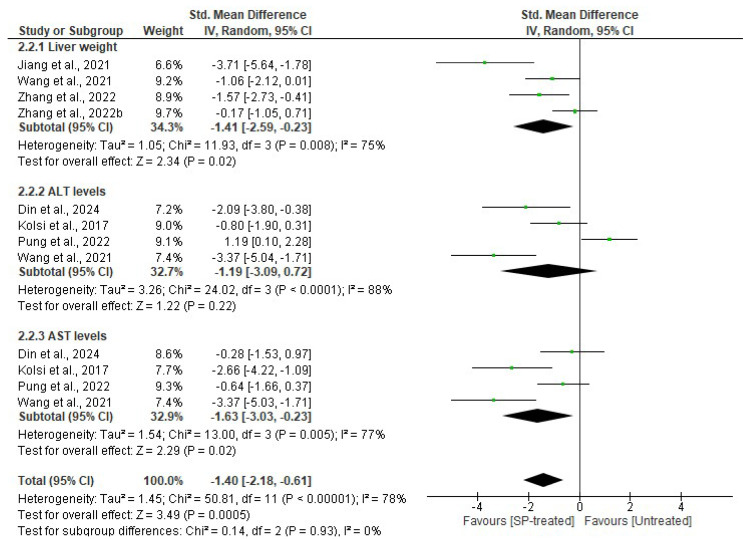
Effects of seaweed-derived SPs on liver weight, serum ALT, and serum AST levels [20,21,23,25,26,27,28]. The forest plot shows the impact of seaweed-derived SP treatment on liver weight, ALT, and AST compared to their corresponding untreated groups. The values of *p* ≤ 0.05 were considered significantly different from each other.

Studies that evaluated the effects of seaweed-derived SPs on liver weight and liver function enzymes, ALT and AST, in comparison to their respective untreated groups were included in the analysis (Figure 7). There was a significant reduction in the liver weight (*p* = 0.02) (*n* = 33 SMD = −1.41, 95% CI = −2.59, −0.23) and AST levels (*p* = 0.02) (*n* = 28 SMD = −1.63, 95% CI = −3.03, −0.23) in the SP-treated groups compared to their respective untreated groups. However, there was no significant difference (*p* = 0.22) observed in the serum ALT (*n* = 23 SMD = −1.19, 95% CI = −3.09, −0.72) levels in the SP-treated groups compared to the corresponding untreated groups. Significant heterogeneity was observed in all included studies that assessed liver weight (*I*^2^ = 75%, df = 3, *p* = 0.008), ALT (*I*^2^ = 88%, df = 3, *p* < 0.0001), and AST (*I*^2^ = 77%, df = 3, *p* = 0.005) (Figure 7).

#### 2.2.3. Effects of Seaweed-Derived SPs on Serum Insulin

**Figure 8:**
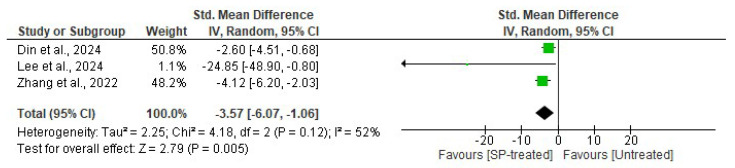
Effects of seaweed-derived SP treatment on serum insulin compared to untreated groups [18,20,27]. The forest plot shows the impact of seaweed-derived SP treatment on an obesity hormone, insulin, compared to the untreated group. The values of *p* ≤ 0.05 were considered significantly different from each other.

Hyperinsulinemia is a common phenomenon in obese individuals or obese animals. Three studies evaluated the effects of seaweed-derived SPs on obesity-related hormones, namely insulin, in comparison to an untreated group. The analysis revealed a significant (*p* = 0.005) reduction in serum insulin levels (*n* = 15 SMD = −3.57, 95% CI = −6.07, −1.06) in the SP-treated groups compared to their respective untreated groups, as shown in Figure 8. Additionally, there was no significant heterogeneity observed in the included studies (*I*^2^ = 52%, df = 2, *p* = 0.12).

#### 2.2.4. Effect of Seaweed-Derived SPs on an Inflammatory Biomarker, TNF-α

**Figure 9:**
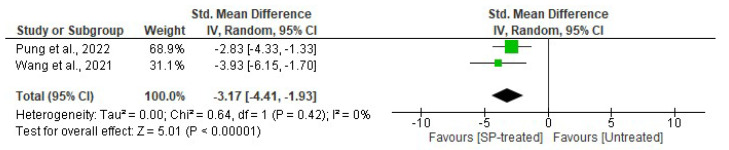
Effects of seaweed-derived SPs on an inflammatory biomarker, TNF-α [25,26]. The forest plot shows the impact of seaweed-derived SP treatment on serum TNF-α compared to the untreated group. The values of *p* ≤ 0.05 were considered significantly different from each other.

Two studies compared the effects of seaweed-derived SP treatment on serum TNF-α levels compared to an untreated group. A significant (*p* < 0.00001) reduction in serum TNF-α was observed in the serum of seaweed-derived SP-treated groups compared to the respective untreated groups (*n* = 14 SMD = −3.17, 95% CI = −4.41, −1.93), with no significant heterogeneity (*I*^2^ = 0%, df = 1, *p* = 0.42) between the studies included, as shown in Figure 9.

#### 2.2.5. Effects of Seaweed-Derived SPs on the Serum Lipid Profile

**Figure 10:**
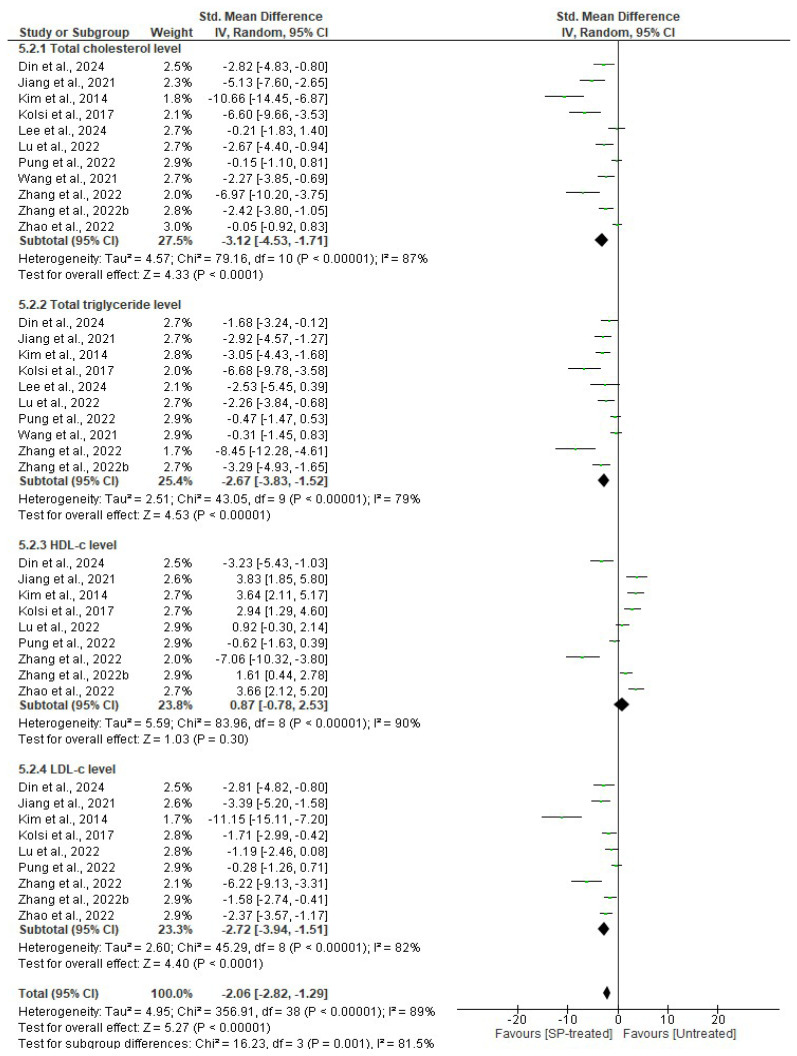
Effects of seaweed-derived SPs on the serum lipid profile, including total cholesterol, triglycerides, LDL-c, and HDL-c [18,20,21,22,23,24,25,26,27,28,29]. The forest plot shows the impact of seaweed-derived SP treatment on the serum lipid profile compared to the untreated group.

The analysis revealed a significant reduction in serum total cholesterol (*n* = 77 SMD = −3.12 95% CI = −4.53, −1.71), triglycerides (*n* = 67 SMD = −2.67, 95% CI = −3.83, −1.52), and LDL-c levels (*n* = 68 SMD = −2.72, 95% CI = −3.94, −1.51) in the seaweed-derived SP-treated groups compared to the corresponding untreated groups. However, the effects were significantly better for the reduction in serum triglycerides (*p* < 0.00001) compared to total cholesterol and LDL-c (*p* < 0.0001). Additionally, there was no significant difference (*p* = 0.30) observed in the serum HDL-c levels (*n* = 68 SMD = 0.87, 95% CI = −0.78, 2.53) between the SP-treated groups in comparison to the corresponding untreated groups. However, there were significant differences observed in terms of the heterogeneity of the studies for total cholesterol (*I*^2^ = 87%, df = 10, *p* < 0.00001), triglycerides (*I*^2^ = 79%, df = 9, *p* < 0.00001), HDL-c (*I*^2^ = 90%, df = 8, *p* < 0.00001), and LDL-c (*I*^2^ = 82%, df = 8, *p* < 0.00001) (Figure 10).

## 3. Discussion

Sulphated polysaccharides (SPs) are a complex group of carbohydrates containing sulphate groups. They are present in green (Chlorophyceae), red (Rhodophyta), and brown (Phaeophyceae) seaweeds, and are referred to as ulvans, carrageenan, and fucoidans, respectively [15]. Sulphated polysaccharides play a crucial role in maintaining the structural integrity of the seaweed’s cell wall by maintaining stability and preventing dehydration. They exhibit variability in composition and structure depending on factors such as seaweed species, geographic location, harvesting season, anatomical regions, and the type of extraction used [30]. Furthermore, SPs isolated from seaweeds have been shown to have antioxidant, anti-viral, anti-inflammatory, anti-allergic, anti-tumour, anti-obesity, anti-coagulant, anti-hepatopathy, and anti-nephropathy properties [31]. 

**Figure 11:**
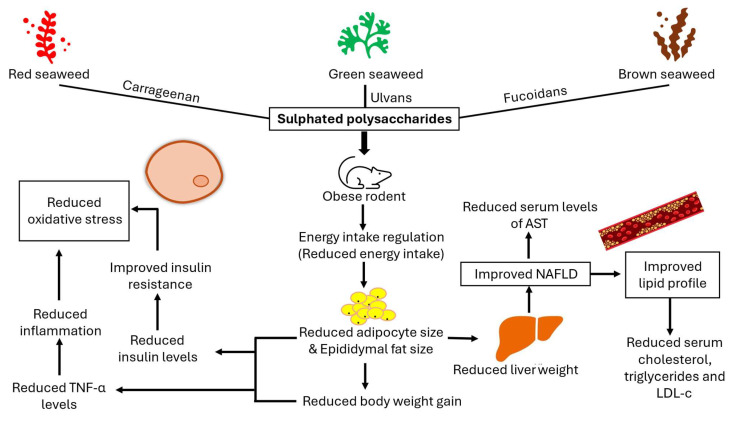
Proposed summary of anti-obesity potential of SPs isolated from seaweeds in rodents.

Many studies have demonstrated the anti-obesity effects of SPs through animal model studies, and many studies reported that the administration of seaweed-derived SPs to obese animal models improved obesity and associated parameters [18,20,21,22,23,24,25,26,27,28,29]. The present study provides a systematic review and meta-analysis of studies on the ameliorative effects of seaweed-derived SPs on obesity and its related parameters, including food intake, body weight gain, adipose size, liver weight and liver function enzymes, inflammatory biomarkers, insulin, and the lipid profile using in vivo animal model-based studies. This study indicates a direction as to whether further investigations of the anti-obesity potential of seaweed-derived SPs are necessary and if they can be further isolated to be tested in humans via clinical trials. The summary of the anti-obesity effects exerted by SPs from seaweeds as per eligible studies used in this study are illustrated in Figure 11.

Hyperphagia (overeating) accompanied by a sedentary lifestyle are some significant risk factors of obesity. Leptin is an important hormone that maintains energy balance by regulating energy intake and expenditure by signalling the brain about the body’s energy stores [32]. Leptin deficiency and leptin resistance is common in obesity, which explains the reduced satiety, hyperphagia, and increased body weight in obese individuals. Several studies have evaluated the effects of seaweed-derived SPs on food intake in obese animal models [20,22,23,25,27,29]. Although the data were not significantly different, the obese animals administered with seaweed-derived SPs exhibited lower food intake compared to the obese animals without treatment (Figure 1 and Figure 6). This suggests that SPs from seaweeds may have ameliorative effects on leptin deficiency and leptin resistance [33].

Obesity-related fat accumulation causes white adipose tissue to expand, resulting in adipose hypertrophy and the formation of new adipocytes, known as hyperplasia [34]. The body weight gain observed in obesity is associated with this enlargement and formation of new adipocytes. Notably, epididymal fat depots exhibit significant growth during the early stages of weight gain. Consequently, many animal studies were focused on epididymal fat when investigating the weight gain-related parameters. This highlights the importance of considering body weight gain, epididymal fat size, and adipocyte size as crucial factors in obesity studies. Many studies have evaluated the protective effects of SPs derived from seaweeds on body weight gain, epididymal fat size, and adipocyte size using animal models [18,20,21,22,23,24,25,26,27,28,29]. Obese animals demonstrated a significant increase in body weight gain, as well as epididymal fat padding and adipocyte size. However, the obese animals that were supplemented with SPs from seaweeds showed significantly lower body weight gain, epididymal fat, and adipocyte hypertrophy than the obese animals without treatment (Figure 1 and Figure 6). This may be attributed to a reduced food intake that is observed in SP-treated animals, which further suggests that SPs from seaweeds may be great candidates to treat obesity.

Obesity causes chronic inflammation, which leads to many disorders including oxidative stress, insulin resistance, and diabetes [35]. Increased inflammation levels in obesity are indicated by elevated levels of serum cytokines including TNF-α and IL-6 [6]. Tumour necrosis factor-α (TNF-α) is a proinflammatory cytokine that plays a crucial role in adipose tissue, influencing metabolism and insulin signalling. Reports have shown a positive correlation between TNF-α and obesity and a negative correlation with weight loss, suggesting a link between TNF-α and adiposity [6,7]. Obese rodents have elevated levels of TNF-α, prompting research into the potential of seaweed-derived SPs to reduce this cytokine. Pung et al. [25] and Wang et al. [26] reported a significant suppression of serum proinflammatory cytokine TNF-α in obese animals that were administered with seaweed-derived SPs. These findings demonstrate that SPs from seaweeds can reduce the serum and adipose low-grade inflammation that is usually observed in obese individuals (Figure 3 and Figure 9). Tumour necrosis factor-α is a key regulator of insulin resistance that presents in obese individuals by downregulating the expression of glucose transporter-4 (GLUT-4) and is the link between obesity and insulin resistance [36]. Obesity is associated with high levels of insulin (hyperinsulinemia), which stems from insulin resistance caused by an increased cytokine release. Studies have shown elevated insulin levels in obese rodents. However, supplementing the animals with seaweed-derived SPs significantly reduced the insulin levels in obese animals (Figure 8) [18,20,27]. These findings may be attributable to the anti-inflammatory properties of seaweed-derived SPs, leading to the upregulation of GLUT-4 and reversing obesity-associated insulin resistance.

The liver is an important organ, as it plays a crucial role in regulating blood glucose homeostasis in response to hormones, insulin, and glucagon [37]. The excessive accumulation of fats during obesity can cause a buildup of fatty deposits in the liver, leading to non-alcoholic fatty liver disease (NAFLD), which affects the regulation of glucose. Non- alcoholic liver disease leads to an increased liver size, which is contributed to by fat depots [38]. The enzymes alanine transaminase (ALT) and aspartate transaminase (AST) are crucial in amino acid metabolism, and their serum levels serve as important indicators of liver health and liver disease. An elevation in the serum AST and ALT levels indicate liver injury [39]. Furthermore, the levels of these enzymes have been reported to increase with obesity [40]. Consequently, assessments of liver weight and serum ALT and AST are essential for evaluating hepatic health. According to several studies [20,21,23,25,26,28,29], obese rodents have a higher liver weight, as well as higher levels of liver enzymes, ALT and AST, in the serum. However, administering SPs derived from seaweeds alleviated these effects by reducing liver weight and lowering serum AST levels in obese animals with no significant difference for serum ALT levels (Figure 2 and Figure 7). These findings highlight the potential of seaweed-derived SPs to module fat accumulation in obesity, thereby preventing the formation of lipid deposition in the liver. Furthermore, the SPs from seaweeds reverse liver injury and restore normal hepatic function, as evidenced by reduced ALT and AST liver enzymes in the SP-treated group, only significantly so for the AST level (Figure 2 and Figure 7). This also reveals that the impact of liver function enzymes, ALT and AST, on the pathogenesis of obesity may not be the same.

The liver plays a pivotal role in lipid metabolism, serving as a primary site for the absorption, synthesis, and secretion of lipoproteins into the circulatory system [41]. The distinct functional capabilities of the liver enable it to regulate the uptake, processing, and distribution of lipids, thereby influencing lipid homeostasis and overall metabolic health. HDL-c is produced in the liver and helps to remove excess cholesterol in the peripheral tissues and transport it to the liver for excretion [42]. Low levels of HDL-c in obese and non-obese conditions are associated with cardiovascular disease [43]. Furthermore, the liver produces LDL-c, total cholesterol, and triglycerides, and it is responsible for maintaining a balance between their production and secretion [38]. High levels of cholesterol, triglycerides, and LDL-c are associated with an increased risk of several health problems, including cardiovascular diseases, fatty liver disease, and diabetes mellitus [41,42]. Studies have shown that obese rodents have elevated levels of LDL-c, triglycerides, and cholesterol, accompanied by low levels of HDL-c [18,20,21,22,23,24,25,26,27,28,29]. However, intervention with SPs from seaweeds ameliorated these effects, as evidenced by reduced levels of LDL-c, triglycerides, and total cholesterol; however, no significant difference was observed for HDL-c levels (Figure 5 and Figure 10). These findings suggests that SPs from seaweeds exert beneficial effects on lipid metabolism, thereby requiring further investigation into their potential therapeutic applications in the management of dyslipidemia and related metabolic disorders.

## 4. Materials and Methods

**Figure 12:**
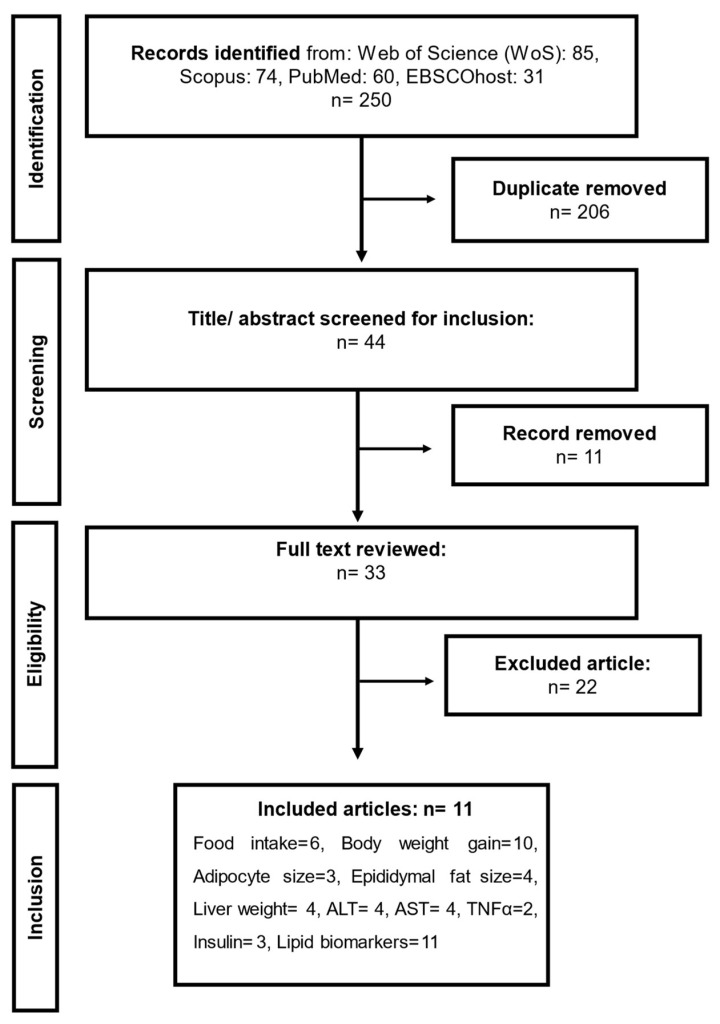
The flow chart showing preclinical (in vivo) studies included in the meta-analysis that evaluated the effect of seaweed-derived SPs on obesity and related parameters.

The methodology employed in this review was specified in advance and documented in a protocol registered on the Prospective Register of Systematic Reviews (PROSPERO) under registration ID CRD42024590635. This systematic review and meta-analysis were developed and reported in accordance with the Preferred Reporting Items for Systematic Review and Meta-analysis Protocol guidelines and abstract checklist (PRISMA-P) [44]. The PRISMA-P checklist was completed to optimize the reliability of the protocol used, as shown in Figure 12.

### 4.1. Study Design and Identification of Articles

Preclinical studies, including in vivo studies that evaluated the effects of seaweed-derived sulphated polysaccharides (SPs) on obesity, were systematically searched on EBSCOhost, MEDLINE via PubMed, Scopus, and Web of Science (WoS). The search strategy used a combination of meSH terms and keywords, with the search terms divided into three components, namely (1) intervention, (2) type of study (model of study), and (3) obesity component. The topic (title/abstract) search was carried out using keywords or their combinations such as ‘Seaweed sulphated polysaccharides’ OR ‘Seaweed-derived sulphated polysaccharide’ OR ‘Seaweed sulphated polysaccharides OR ‘Seaweed-derived sulphated polysaccharides’ OR ‘Brown seaweeds fucoidans’ OR ‘‘Green seaweeds ulvans’ OR ‘Red seaweeds carrageenan’ AND ‘Preclinical studies’ OR ‘Experimental models’ OR ‘In vivo’ OR ‘Rats’ OR ‘Rat’ OR ‘Mice’ OR ‘Mouse’ OR ‘Obesity model’ OR ‘Rodents’ OR ‘Rodent’ OR ‘Animal model’ AND ‘Obesity’ OR ‘Inflammation’ OR ‘Hyperlipidaemia’ OR ‘Metabolic diseases’ OR ‘Body weight’ OR ‘Dyslipidaemia’ with no regional, spatial-temporal, or lingual limits. The search strategy was adjusted according to each database specification for the most optimal recovery. Furthermore, new records were tracked using an email alert system until the final analysis. The search was carried out by two of the authors (H.I and N.H), who imported and de-duplicated all the records in Endnote 20 and in Microsoft Excel. Two authors of this study (N.P.H and V.N.C) screened the titles and abstracts of the articles using the set inclusion criteria. Full article reviews were conducted on articles that met the criteria based on the title and abstract review by two authors (V.N.C and S.N.M) and were selected based on the variables of interest. The last author (M.S.I) provided necessary guidance and supervision and thoroughly reviewed the entire manuscript before submission. The retrieval of records and processing were performed in accordance with PRISMA, as shown in Figure 1.

### 4.2. Study Design Eligibility Criteria

This systematic review and meta-analysis included in vivo (randomized and non-randomized) studies that assessed the effects of SPs, particularly those extracted from seaweeds, on obesity and related parameters. Due to the lack of clinical trials, only preclinical studies (in vivo) that assessed some or all of the parameters such as food intake, body weight gain, epididymal fat size, adipocyte size, liver weight, serum alanine transaminase (ALT), aspartate transaminase (AST), insulin and tumour necrosis factor-α (TNF-α), and the lipid profile (total cholesterol, triglycerides, high-density lipoprotein (HDL), and low-density lipoprotein (LDL)) and published in English were included in the study. Studies without a control group were excluded to provide a balance in the baseline of the variables. The untreated group was the control (placebo), the untreated control was those that were in the diseased group but not treated, and the SP-treated group was the diseased group that was treated with SPs. Furthermore, studies that sufficiently demonstrated the relevance of the used assays for the desired outcomes while also providing measurable results were included in this review. The studies that used one or a combination of in vitro, ex vivo, in silico, and clinical trial designs were excluded from this review to stick with the preclinical studies.

### 4.3. In Vivo Eligibility Criteria

Only in vivo studies that evaluated the parameters of interest were included. For studies with multiple doses, the results of the highest or most effective doses were included. All obesity models (high-fat–high-carbohydrate diet (HF-HC) or chemically induced and genetic and surgically manipulated), sex, strain, age, and the species of animals were included in this review. Additionally, the animal model that closely resembled certain factors of the underlying cause of human obesity, such as being overweight and obese (determined by BMI) was included in this review. All other in vivo studies evaluating other diseases and parameters, except for obesity, were excluded from this review.

### 4.4. Intervention Eligibility Criteria

This study evaluated the efficacy and safety of seaweed-derived SPs. Only studies that performed an intervention of SPs on animals that were induced with obesity were included. Studies that used fractions of or crude SPs and combination therapy, where the SP dose was combined with another component, were not included in this study. Furthermore, preclinical studies involving any other polysaccharide derivatives were not included.

### 4.5. Comparison

To compare between the untreated and experimental group, only studies with the following groups were included: control, obesity-induced group without treatment, and obesity-induced group treated with seaweed-derived SPs.

### 4.6. Statistical Analysis 

The data of interest were extracted from the eligible studies and combined for meta-analysis, using ReviewManager (Revman) version 5.4 (Nordic Cochrane Centre, Copenhagen, Denmark). The data, comprising the mean, number of observations, and standard deviation (see supplemental materials, Tables S1–S13) related to the seaweed-derived SP-treated obese and control group and the seaweed-derived SP-treated obese and untreated obese group were analyzed using a random effects model and the inverse variance method. As continuous outcomes were expected, the effect measure used was the standard mean difference (SMD) with an associated 95% confidence interval (95% CI) to provide a precise estimate of the treatment effect. The random effects model accounted for heterogeneity (*I*^2^) between studies to ensure the reliability of data analysis. To assess statistical heterogeneity, a visual inspection of the forest plots to detect any apparent outliers or deviations from the overall trend was carried out. Additionally, statistical heterogeneity was quantified, with a significance level set at *p* < 0.05. The degree of heterogeneity was evaluated using the *I*^2^ statistic and interpreted as follows: 0% indicates no evidence of heterogeneity, 30–60% suggests moderate heterogeneity, and 75–100% indicates high heterogeneity, suggesting significant inconsistencies across the studies. The meta-analysis was conducted on studies with two or more of the following outcomes: food intake, body weight gain, epididymal fat size, adipocyte size, liver weight, ALT, AST, serum insulin and TNF-α, total cholesterol, total triglycerides, HDL-c, and LDL-c.

## 5. Strengths and Limitations

This study was conducted by performing a comprehensive systematic and meta-analysis approach following to PRISMA guidelines to investigate the potential of seaweed-derived sulphated polysaccharides (SPs) in alleviating obesity and related parameters. A comprehensive literature search was conducted by three independent authors, minimizing errors and ensuring a thorough analysis. By focusing on purified SPs, the study isolated their effects and excluded studies that assessed crude extracts, fractions, or the combinations of SPs with other components. This approach enabled a detailed assessment of multiple obesity-related parameters, providing valuable insights into the potential benefits of seaweed-derived SPs. However, the study has some limitations, including the literature search being restricted to major electronic databases, potentially overlooking relevant studies in other databases or grey studies. Additionally, the inclusion criteria were limited to English language-based articles focusing on obesity, excluding studies on other diseases and potentially introducing language bias. The study only assessed obesity-related parameters with multiple studies, leading to a selective reporting of outcomes. Furthermore, the focus on purified SPs from seaweeds may not represent all types of SPs, such as fucoidans, carrageenans, or ulvans, which could impact the study’s overall quality. These limitations highlight the need for further research on the anti-obesity potential of not only seaweed-derived SPs but also from other possible sources with articles published in multiple languages. Additionally, the effects of SPs on gut microbiota and short-chain fatty acid production from gut fermentation need to be considered in future studies.

## 6. Implications for Future Research

This systematic review suggests that seaweed-derived sulphated polysaccharides (SPs) exhibit anti-obesity potential, although the meta-analysis did not provide conclusive evidence for all but most of the obesity-related parameters. Further research is necessary to substantiate the anti-obesity effects of SPs. To achieve this, investigations should be conducted on various types of SPs (ulvans, carrageenans, and fucoidans) from different seaweed species and other sources, using human subjects to examine their effects on obesity-related parameters in humans. Furthermore, elucidating the mechanisms of action, including their impact on gut microbiota and SCFA production from gut fermentation, would provide valuable insights. Standardized extraction and purification methods for SPs must be developed to ensure consistency in research findings. Moreover, long-term studies are also essential to assess the sustainability of SPs’ anti-obesity effects, as well as to explore the potential of SPs in preventing related diseases, such as diabetes and cardiovascular disease, which may reveal new therapeutic avenues. Additionally, investigating the synergistic effects of combining seaweed-derived SPs with other anti-obesity compounds could lead to enhancing the efficacy of SPs as well.

## 7. Conclusions

This study assessed the potentials of seaweed-derived sulphated polysaccharides (SPs) to modulate obesity-related parameters, including food intake, body weight gain, lipid accumulation, inflammation, insulin resistance, hepatic injury, and lipid profiles. The systematic findings revealed that seaweed-derived SPs ameliorated obesity parameters by decreasing body weight gain and reducing epididymal fat and adipocyte size. Additionally, SPs from seaweeds mitigated inflammation and insulin resistance, as evidenced by reduced TNF-α levels and serum insulin concentrations, particularly in obese animal models. Furthermore, treatment with seaweed-derived SPs resulted in a decreased liver weight and some relevant enzymes, as well as reduced LDL-c, triglycerides, and cholesterol levels in obese rats. However, the meta-analysis indicated that these findings were not statistically significant for liver a function enzyme, ALT, and serum HDL-c, highlighting the need for further research to provide conclusive evidence of the efficacy of seaweed-derived SPs on obesity and its associated parameters. Further investigations into the ameliorative effects of seaweed-derived SPs, including the isolation of specific SPs and examination of additional obesity biomarkers and enzymes in human subjects, are required to ascertain the results of the preclinical studies.

## Figures and Tables

**Table 1:** Characteristics of eligible studies and their outcomes.

References	n	Animal Models	Seaweed Species	Route of Administration	Outcomes
Din et al., [20]	25	Sprague Dawley rats ^a^	*Sargassum binderi*	Orally	1, 7, 8, 9, 10, 11, 12, 13
Jiang et al., [21]	40	BALB/c mice ^a^	*Undaria pinnatifida*	Oral gavage	2, 3, 4, 5, 10, 11, 12, 13
Kim et al., [22]	30	C57BL/6 mice ^a^	*brown seaweed*	-	1, 2, 4, 10, 11, 12, 13
Kolsi et al., [23]	28	Wistar rats ^a^	*Sargassum vulgare*	Gastric gavage	1, 2, 6, 7, 10, 11, 12, 13
Lee et al., [18]	50	C57 BL/6J mice ^a^	*Ecklonia cava*	Diet supplemented	2, 8, 10, 11, 12
Lu et al., [24]	40	C57BL/6J mice ^a^	*Laminaria japonica*	Oral gavage	1, 2, 4, 10, 11, 12, 13
Pung et al., [25]	55	C57BL/6 mice ^a^	*Ulva prolifera*	Diet supplemented	1, 2, 7, 8, 9, 10, 11, 12, 13
Wang et al., [26]	32	C57BL/6 mice ^a^	*Fucus vesiculosus*	Intraperitoneally	2, 5, 6, 7, 9, 10, 11
Zhang et al., [27]	49	C57BL/6J mice ^a^	*Laminaria japonica*	Intragastric gavage	1, 2, 3, 8, 10, 11, 12, 13
Zhang et al., [28]	40	BALB/c mice ^a^	*Undaria pinnatifida*	Oral gavage	2, 3, 4, 5, 10, 11, 12, 13
Zhao et al., [29]	40	C57BL/6J mice ^a^	*Enteromorpha polifera*	Intragastric gavage	2, 5, 10, 12, 13

1—food intake; 2—body weight gain; 3—adipocyte size; 4—epididymal fat size; 5—liver weight; 6—ALT; 7—AST; 8—serum insulin; 9—serum TNF-α; 10—total cholesterol; 11—total triglyceride; 12—HDL-c; and 13—LDL-c. ^a^ High-fat diet (HFD)-induced obesity; (-)—not specified.

## Data Availability

Not applicable, since this is a review article and meta-analysis.

## Supplementary Materials

The following supporting information can be downloaded at: https://www.mdpi.com/article/10.3390/md22120528/s1, in which the raw data of the meta-analysis including: mean, standard deviation and size are included for each parameter. Table S1: Data on food intake of SP-treated, control and untreated groups. Table S2: Data on body weight gain of the SP-treated, control and untreated groups. Table S3: Data on epididymal fat size of the SP-treated, control and untreated group. Table S4: Data on adipocyte size of the SP-treated , control and untreated groups. Table S5: Data on adipocyte size of the SP-treated, control and untreated groups. Table S6: Data on ALT levels of the SP-treated, control and untreated groups. Table S7: Data on AST levels of the SP-treated, control and untreated groups. Table S8: Data on TNF-a levels of the SP-treated, control and untreated groups. Table S9: Data on serum insulin levels of the SP-treated, control and untreated groups. Table S10: Data on TC levels of the SP-treated, control and untreated groups. Table S11: Data on TG levels of the SP-treated group, control group and untreated groups. Table S12: Data on HDL-c levels of the SP-treated, control and untreated groups.; and Table S13: Data on LDL-c levels of the SP-treated, control and untreated groups.
